# Generalized Acquired Cutis Laxa Associated with Monoclonal Gammopathy of Dermatological Significance

**DOI:** 10.1155/2020/7480607

**Published:** 2020-02-12

**Authors:** Sophia Z. Shalhout, Myrna R. Nahas, Reed E. Drews, David M. Miller

**Affiliations:** ^1^Division of Hematology/Oncology and Department of Dermatology, Massachusetts General Hospital, Harvard Medical School, Boston, MA 02114, USA; ^2^Division of Hematology/Oncology, Beth Israel Deaconess Medical Center, Harvard Medical School, Boston, MA 02215, USA

## Abstract

**Background:**

Cutis laxa is a rare dermatosis that is inherited or acquired and clinically features loose, wrinkled, and redundant skin with decreased elasticity. This heterogeneous connective tissue disorder may be localized or generalized, with or without internal manifestations. Generalized cutis laxa often has a cephalocaudal progression and is attributed to inflammatory cutaneous eruptions, medications, and infections. Cutis laxa is also associated with several other conditions including rheumatoid arthritis, systemic lupus erythematosus, and plasma-cell dyscrasias. *Case Presentation*. We report an unusual case of a 35-year-old male with progression of generalized acquired cutis laxa and vasculitis that occurred over a period of one year. No cutaneous inflammatory eruption preceded or accompanied his decreased skin elasticity, and a biopsy of the skin showed elastolysis. His cutaneous manifestation led to systemic evaluation and an eventual diagnosis of smoldering multiple myeloma accompanied by aortitis and anemia. His myeloma and vasculitis were successfully treated with cyclophosphamide, bortezomib, and dexamethasone and high-dose prednisone, respectively, with no improvement to his cutis laxa.

**Conclusions:**

The presence of monoclonal gammopathy is strongly associated with several dermatological entities such as acquired cutis laxa. We propose a new term for the dermatological manifestations caused by paraproteinemia: monoclonal gammopathy of dermatological significance, or MGODS, and stress the evaluation of an underlying gammopathy in the setting of certain dermatologic conditions, including scleromyxedema and amyloidosis. We present a case of a newly acquired cutis laxa secondary to plasma-cell dyscrasias that exemplifies MGODS, alongside a brief literature review, and underscore the clinical relevance of monoclonal gammopathies of dermatological significance.

## 1. Background

Cutis laxa (CL), or elastolysis, is a rare heterogeneous dermatosis with several etiologies. CL is typically inherited as a dominant, recessive, or X-linked recessive condition. Clinically, CL is characterized by loosely hanging, pendulous skin folds, resulting in the appearance of premature aging. Histologically, degeneration of the dermal elastic fibers is observed. Internal organ involvement is often seen in patients with CL and can affect the pulmonary, gastrointestinal, urogenital, and cardiovascular systems. Prognosis of CL may vary and largely depends on the implicated gene mutation as well as the extent of systemic involvement. It may range from a fatal outcome in certain cases of inherited CL to a normal life expectancy in the less severe forms. CL is a progressive disorder with insidious onset that worsens with age. In rare cases, CL may be acquired *de novo* or associated with preceding cutaneous inflammatory eruptions with adult-onset of disease. CL is also associated with infections, drug hypersensitivity reactions, and plasma-cell dyscrasias. We report a rare case of generalized acquired CL and aortitis, without preceding inflammatory lesions or eruptions, most likely secondary to multiple myeloma. The presence of monoclonal gammopathy is strongly associated with several dermatological entities and cutaneous manifestations. We suggest these should be referred to as monoclonal gammopathy of dermatological significance, or MGODS, and stress the evaluation of an underlying gammopathy in the setting of several well-established dermatological features. We present a case of MGODS and provide a brief literature review of other cases of acquired cutis laxa associated with monoclonal gammopathy.

## 2. Case Presentation

A 35-year-old male noticed the progression of loosening and thinning of his skin over the period of one year. These areas had been asymptomatic with no preceding inflammatory dermatosis. The patient's past medical history included hypertension and long-standing anemia of uncertain etiology.

**Figure 1:**
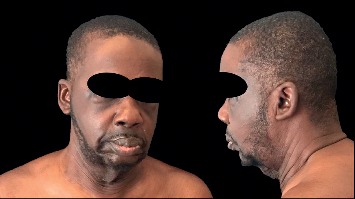
At the age of 35: appearance of premature aging; wrinkly face with profound laxity notable in the periocular skin and neck region.

**Figure 2:**
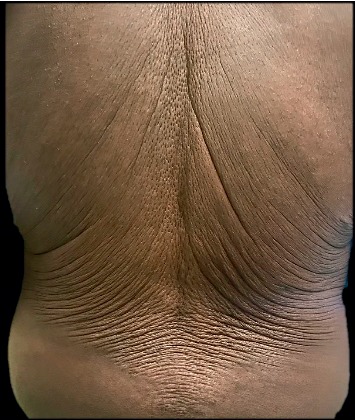
Back with evident loose, wrinkly, redundant skin folds.

On physical exam, profound laxity of the periocular skin, neck, axillary, and back was appreciated (Figures 1 and 2). The clinical presentation was suggestive of an acquired cutis laxa. A biopsy of involved skin demonstrated sparse superficial perivascular lymphocytic inflammation with rare giant cells. An elastic stain showed a decrease in the elastic fibers in the reticular dermis compared to a biopsy of uninvolved skin; no significant changes in the dermal collagen were appreciated on Masson's Trichome staining. Findings consistent with granulomatous slack skin were absent. In aggregate, the clinical and pathological presentation were consistent with an acquired cutis laxa (ACL).

**Figure 3:**
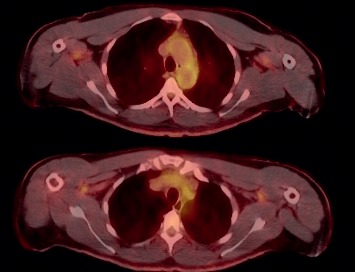
PET-CT shows FDG avidity of the vascular walls of the aorta.

Serology work-up for autoimmune connective tissue disease was negative for rheumatoid factors, antineutrophil cytoplasm antibodies, and anti-nuclear antibodies. Chromosomal microarray analysis performed on DNA extracted from a peripheral blood specimen returned normal findings. Anemia work-up demonstrated normal iron stores, normal renal function, and decreased reticulocyte count. He was found to have an elevated C-reactive protein (CRP) and erythrocyte sedimentation rate (ESR), and an IgG-kappa paraprotein of 1.36 g/dL. The patient was initially diagnosed with monoclonal gammopathy of undetermined significance (MGUS) and anemia of inflammation. Due to suspicion of a connection between his MGUS and cutis laxa, further evaluation revealed an abnormal kappa/lambda free-light chain ratio of 5.86. A PET-CT (Positron emission tomography-computed tomography) did not reveal evidence of skeletal lytic lesions or extraosseous findings of plasmacytoma. However, mild fluorodeoxyglucose avidity of the vascular walls of the aorta and branching vessels was seen, and in the setting of elevated CRP and ESR, was consistent with aortitis and mild vasculitis (Figure 3). Subsequent bone marrow biopsy demonstrated 20% plasma cells in the bone marrow core and 13% of aspirate.

In the setting of end-organ dysfunction presumed to be secondary to a plasma-cell dyscrasia (cutis laxa and vasculitis), the patient was started on CyBorD (cyclophosphamide, bortezomib, dexamethasone). His second cycle was delayed due to a left inguinal herniorrhaphy with unremarkable wound healing. CyBorD was tolerated well by the patient. Upon completion of his sixth cycle of CyBorD, a PET-CT revealed continued aortitis, a hiatal hernia, umbilical hernia, and small bowel-containing right inguinal hernia. The level of monoclonal IgG-kappa protein continued to decline (280 mg/dL), but C-reactive protein levels remained elevated. He was given high-dose oral prednisone, 60 mg/d, to treat his vasculitis which resulted in near normal C-reactive protein levels. His cutis laxa did not improve in response to corticosteroids and immunosuppressive therapy. However, at one-year posttreatment, his cutis laxa did not clinically appear to have progressed.

## 3. Discussion and Conclusions

ACL is extremely rare and can be generalized or localized. Several conditions warrant discussion in the differential diagnosis of the skin manifestations in this patient, including anetoderma, and mid-dermal elastolysis. Anetoderma usually involves the trunk and extremities and consists of well-circumscribed atrophic or depressed patches or saccular outpouchings of wrinkled skin. The histological finding of anetoderma is loss of elastic fibers in the papillary and reticular dermis. Mid-dermal elastolysis is clinically characterized by well-circumscribed fine wrinkles or perifollicular papular protrusions usually involving the trunk and upper extremities. Mid-dermal elastolysis is histologically characterized by focal loss of elastic fibers in the mid-dermis.

ACL has been associated with inflammatory dermatoses, including urticaria, systemic lupus erythematosus, dermatitis herpetiformis, and amyloidosis. Cases of ACL due to medication exposure and arthropod bite reactions have been reported [1, 2]. Our patient is most consistent with reports of ACL associated with underlying hematological disorders including multiple myeloma, plasma-cell dyscrasia, and heavy chain deposition disease [3, 4]. The clinical features, treatment, and outcomes of previous cases reported in the literature of ACL associated with plasma-cell dyscrasias and monoclonal gammopathies are summarized in Table 1. There are reports of at least 23 cases, with ∼20% reporting acral localization of cutis laxa, ∼30% reporting a diagnosis of multiple myeloma associated with amyloidosis, and ∼50% describing a preceding cutaneous process. Very few reports describe stabilization of cutis laxa following systemic therapy administered to treat the underlying monoclonal gammopathy. Unlike other connective tissue disorders, ACL has not been associated with vascular fragility, and surgery is not contraindicated since ACL is not thought to affect wound healing. Early management with plastic surgical procedures such as rhytidectomy can be beneficial to mitigate symptoms. However, serial reconstructive procedures are usually required since CL often progresses with time.

Systemic elastolysis has been reported in adult-onset ACL. The most significant internal organs often involved are associated with pulmonary, cardiovascular (i.e., heart failure, ectasia of the aorta, and aortic aneurysms), gastrointestinal (i.e., diverticula and hernias), and urogenital (i.e., hernias, uterine prolapse, and cystocele) systems [25–27]. It is unclear if the aortitis, large vessel vasculitis, and numerous hernias experienced by this patient were related to the paraprotein and elastolysis [4, 8]. Although the exact pathophysiology is unknown, myeloma-associated immunoglobulin deposition is thought to result in a cell-mediated immune response and promotes phagocytic destruction of elastic fiber [4, 28]. In our patient, we suspect that the paraprotein contributed to decreased skin laxity as well as involvement of the vasculature and gastrointestinal system. There is one case report of congenital cutis laxa associated with aortitis in a 17-month-old child [26]. However, to our knowledge, there are no reports of ACL and aortitis secondary to multiple myeloma.

We propose a new term for the dermatological manifestations caused by paraproteinemia: monoclonal gammopathy of dermatological significance or MGODS. This term encapsulates a variety of diagnoses that present with paraproteins and may have significant cutaneous involvement such as nodular and light chain amyloid, cryoglobulinemia, necrobiotic xanthogranuloma, scleromyxedema, papular mucinosis, and POEMS syndrome [29].  Table 2 provides a nonexhaustive list of well-established conditions associated with monoclonal gammopathy and a summary of pertinent cutaneous findings.

MGODS may present in the setting of an otherwise monoclonal gammopathy of undetermined significance (MGUS). Indeed, in our case, the patient was initially diagnosed with MGUS, but the recognition of the likely ACL representing an MGODS prompted further evaluation, including a bone marrow biopsy, resulting in the diagnosis of smoldering myeloma. Given that skin manifestations are not part of diagnostic criteria for progression beyond MGUS, our case report and the term MGODS highlights the utility of recognizing cutaneous manifestations of monoclonal gammopathies as it may guide evaluation and, in some cases, management. End-organ dysfunction is often the impetus to initiate treatment for plasma-cell dyscrasias, with skeletal, renal, and hematological abnormalities being the most common organs triggering induction of therapy. In certain contexts, however, gammopathies of dermatological significance may necessitate initiation of therapy. The types of skin dysfunction that warrant the induction of systemic treatment are far from agreed upon and often require multidisciplinary consultation.

## Figures and Tables

**Table 1:** Summary of acquired cutis laxa associated with monoclonal gammopathies/plasma-cell dyscrasia.

Reference	Sex, age	Preceding cutaneous eruptions	Clinical features	Associated plasma-cell dyscrasia/monoclonal gammopathy	Treatment	Outcome
Scott et al. [5]	F, 44	Edema of face and neck from hypersensitivity reaction to penicillin	Skin laxity to face and neck, followed by progression to extremities and torso; systemic involvement (gastrointestinal and urogenital)	Multiple myeloma	Surgical repair of hernias/prolapses	Not reported

Ting et al. [6]	F, 45	Intermittent “puffiness” of eyelids	Progressive laxity of the skin starting from the eyelids and spreading gradually to the face, neck, trunk, lungs, rectum, bladder, and perineum	Multiple myeloma	Not reported	Not reported

Frémont et al. [7]	F, 59	None reported	Skin hyperlaxity present for several years	IgG lambda myeloma	Thalidomide	One year after treatment, skin laxity stabilized

Gupta and Helm [8]	F, 62	Denied any prior inflammatory skin disorder or exanthema	Progressive laxity to face, neck, chest, and back; no rectal or vaginal prolapse, emphysema, or cardiac problems detected	Multiple myeloma	Prior to CL onset, patient received vincristine, melphalan, doxorubicin, cyclophosphamide, and prednisone with improvement to hematological disease	Patient was on prednisone during onset of CL; thalidomide gradually increased; but no improvement to cutis laxa was observed

Turner et al. [9]	M, 29	2-year history of asymptomatic urticarial red papules and plaques on the neck, chest, and back lasting days at a time, urticarial vasculitis	Wrinkling and sagging skin on the face, neck, axillae, shoulders, and arms with transverse striae on abdomen, leukocytoclastic vasculitis, and immune complex-mediated glomerulonephritis	IgA myeloma involving kidneys	High-dose methyl prednisolone, and intravenous cyclophosphamide	Initially, all urticarial skin lesions resolved; eventually, renal function deteriorated, and the patient became dependent on dialysis; patient eventually succumbed to his disease

Kluger et al. [10]	M, 40	Chronic urticarial dermatosis of the extremities, mostly involving the hands, progressively worsened, with repeated swelling of the fingers	Acral localization of cutis laxa, joint hyperlaxity, and recurrent neutrophilic urticarial dermatosis	IgA multiple myeloma	Methotrexate, colchicine, hydroxychloroquine, intravenous gamma globulins, and dapsone, oral prednisone	Treatment with oral prednisone resulted in complete remission of the urticarial lesions, with steroid dependence; prevention of the progression in joint laxity or cutis laxa was not achieved

Lavorato et al. [11]	F, 57	Bilateral eyelid hyperchromia, and increase in palpebral volume	Cutaneous laxity in skin folds, bilateral palpebral ptosis, pain and paresthesia, histological and clinical features consistent with primary systemic amyloidosis, cutaneous mucinosis, and acquired cutis laxa	Multiple myeloma associated amyloidosis	Bortezomib and dexamethasone, followed by autologous bone marrow transplantation	“Clinically important dermatological improvement” was achieved

Yoneda et al. [12]	M, 62	None reported, but presented with lumbago and shoulder pain, with a history of severe fatigue and night sweats	Soft, redundant, cutaneous laxity to acral sites on fingertips and soles of feet, lumbago	Myeloma associated amyloidosis	Cyclophosphamide and prednisolone	Treatment decreased hematological disease but the cutis laxa of acral sites progressed; patient eventually succumbed to his disease

Yoneda et al. [12]	M, 71	None reported, but presented with lumbar and back pain, with a history of leg pain, weakness, and night sweats	Soft, loose skin changes to both thumbs	Myeloma associated amyloidosis	Cyclophosphamide	Chemotherapy resulted in a decrease of hematological disease, but cutaneous lesions did not regress; continued follow-up at time of report

Nikko et al. [3]	F, 40	Denied any prior inflammatory skin disorder	Progressive wrinkling, and laxity of the skin on back, chest, abdomen, upper arms, neck, thighs but face was spared	Plasma-cell dyscrasia	None reported	Careful follow-up in case of systemic complication at the time of report.

Lee et al. [13]	F, 54	One-year previous history of easy bruising	Hypopigmented patches with skin laxity, purpura on both flanks, periorbital purpura, lax skin of thumbs; histological, and clinical features consistent with acquired cutis laxa, and primary systemic amyloidosis	Multiple myeloma associated amyloidosis	Bortezomib, thalidomide, dexamethasone followed by autologous peripheral blood stem cell transplant	Slight clinical improvement of skin was noted

Dicker et al. [14]	F, 59	“Puffiness” in fingertips, tender with pressure, tense before resolving to lax skin	Persistent laxity of skin on finger pads, and tongue swelling	Plasma cell dyscrasia	Cyclophosphamide, vincristine, adriamycin, and methylprednisolone	Reduction in size of tongue and a decrease in laxity of skin lesions were achieved

Appiah et al. [15]	F, 64	History of multiple asymptomatic skin lesions in groin and axillae	Flesh-colored papules in axillae and groin, papules with purpura on eyelids, translucent papules and nodules on labia majora, wrinkled loose skin on fingertips	Myeloma associated amyloidosis	Not reported	Not reported

Ferrandiz-Pulido et al. [16]	M, 63	3-month history of asymptomatic skin lesions on ventral aspect of fingers	Soft redundant loose skin on all fingertips and hands	Multiple myeloma-associated amyloidosis	Not reported	Not reported

Silveira et al. [17]	M, 29	Diffuse erythematous plaques, mildly infiltrated papules, and plaques on his trunk	Multiple flaccid erythematous plaques on trunk, neck, and skinfolds with flaccidity of face, axillae, groin, neck, hiatal hernia, eventually developed nephrotic syndrome and acute renal failure	IgG lambda monoclonal gammopathy	Bortezomib, dexamethasone, and thalidomide	No improvement to dermatological lesions observed

New and Callen [4]	M, 48	No preceding cutaneous changes, but he developed erythematous plaques and granuloma annulare like features on his buttocks and lateral hips	4-year history of loose wrinkled skin of his face, chest, upper back, lateral hips, buttocks, and proximal upper extremities	Multiple myeloma	Lenalidomide, dexamethasone, oral pamidronate, and aspirin	With 5 months of therapy, patient had hematological and skeletal lesion stabilization, but his cutis laxa progressed during treatment.

Gonzalez-Ramos et al. [18]	M, 68	3-month history of stable asymptomatic multiple myeloma (progressed after 5 years with MGUS) and 2-month history of hemorrhagic bullae in oral buccal and labial mucosa before presentation	Numerous large hemorrhagic oral bullae, yellowish and purple purpura plaques on eyelids and macroglossia, cutis laxa of axilla and antecubital flexure; clinical and histological features consistent with primary systemic amyloidosis and acquire cutis laxa	Multiple myeloma associated amyloidosis	Intensive chemotherapy	No recurrence of skin lesions; at the time of the report, the patient was awaiting an autologous bone marrow transplant

Tan et al. [19]	M, 50	No preceding skin lesions, and his skin was otherwise asymptomatic, a history of heavy chain deposition disease without evidence of multiple myeloma preceding any cutaneous findings	Weight loss and significant lax skin of axillae, groin, neck, face with periocular involvement with upper lid ptosis and lower lid laxity; subsequent emphysema, leg weakness and peripheral polyneuropathy; no known herniations, diverticula, or aneurysms	Heavy chain deposition disease/monoclonal gammopathy	Prednisone and cyclophosphamide but he presented to dermatological service with end-stage disease, medical therapy for the skin condition was not attempted	There was a transient improvement in renal function; the patient underwent functional blepharoplasty to relieve the ectropion/epiphora

O'Malley et al. [20]	F, 60–69	No preceding cutaneous eruptions; a history of nephrotic syndrome and renal insufficiency due to renal heavy chain deposition disease	Extensive emphysema, lower extremity edema with relapse of her heavy chain deposition disease, marked “hound-dog” facies with lax skin encompassing face, neck, and arms; onset correlating with the time renal involvement was first diagnosed	Heavy chain deposition disease/low-grade plasma-cell neoplasm (complement components on dermal elastic fibers also detected)	Bortezomib and pulse dexamethasone	The patient had subsequent improvement of her nephrotic syndrome and resolution of her acute kidney injury; cutaneous outcome not discussed.

Harrington et al. [21]	F, 38	A history of urticaria, renal insufficiency, heavy chain deposition in heart and kidneys, bilateral lower extremity edema	Excessive wrinkling of the skin that began in the axillae a few years before presentation and progressed to involve her face, extremities, and trunk	Heavy chain deposition disease/monoclonal gammopathy	Lenalidomide with progression of renal failure, requiring temporary dialysis and the discontinuation of this medication; stabilized on bortezomib and dexamethasone	The cutaneous outcome was not described

de Larrea et al. [22]	M, 52	None reported	Cutis laxa of the face, neck, axillae, and groin in the setting of MGUS, renal failure	IgG lambda monoclonal gammopathy	Initially, he was treated with granulocyte CSF but developed alveolar hemorrhage and decreased renal function; he was later treated with bortezomib and oral dexamethasone	A complete hematological response without an increase in bone marrow plasma cells was achieved; he was still on chronic hemodialysis at time of report but his cutis laxa had not progressed, and the patient planned for surgical correction of redundant skin folds.

Majithia et al. [23]	M, 40	None reported; but gave a history of fatigue, shortness of breath, and edema	Loose hanging ear lobes, blepharochalasis, lax nasolabial folds, and increased folds over the neck, axilla, and trunk progressing over two years.	Light and heavy chain deposition disease (LHCDD)	Dexamethasone, cyclophosphamide, and bortezomib	Patient had significant improvement clinically and with hematological disease but was lost to follow-up.

Kim and Klein [24]		She had no history of an inflammatory preceding cutaneous process.	Patient presented with a 10-year history of lax skin with progression in recent years to face, neck, and legs; she was diagnosed with MGUS and eventually light chain multiple myeloma, anemia, and immune-mediated glomerular nephritis; in aggregate, findings were consistent with acquired cutis laxa and systemic lupus erythematosus associated with multiple myeloma	Multiple myeloma and systemic lupus erythematosus	Lenalidomide and low-dose dexamethasone for multiple myeloma. Later, she was treated with bortezomib and dexamethasone, followed by IVIG and danazol	She had a good response to lenalidomide and dexamethasone in terms of reduction of light chain disease, but therapy was discontinued due to cytopenia; excellent response to bortezomib and dexamethasone but discontinued therapy due to cytopenia; IVIG and danazol stabilized her blood counts.

*Current case*	M, 35	None reported	Profound laxity of the periocular skin, neck, axillary and back which progressed over the period of one year; aortitis, several hernias and diverticula.	Multiple myeloma	Cyclophosphamide, bortezomib, dexamethasone, (CyBorD) herniorrhaphy, and high-dose prednisone	His hematological disease stabilized on CyBorD, and high-dose prednisone improved vasculitis; his cutis laxa has not progressed one-year posttreatment.

**Table 2:** Summary of monoclonal gammopathies of dermatological significance.

Disease/Condition	Dermatological presentation	Monoclonal gammopathy
Acquired cutis laxa	Lax, wrinkled, sagging, redundant, inelastic skin	IgG, IgA, light and/or heavy chain deposition disease
Scleromyxedema	Mucinosis, papular and sclerodermoid eruption	IgG (lambda) [30]
Light chain amyloidosis	Purpura, hemorrhagic bullous lesions	IgG (lambda) [31]
Nodular amyloidosis	Papulonodules	IgG, IgA [32]
Waldenstrom macroglobulinemia	Nonspecific ulcers, purpura, and urticarial lesions	IgM [33]
Cryoglobulin vasculitis	Palpable purpura	Type I (IgM) and mixed (IgM and IgG, few polyclonal) [34]
Schnitzler's syndrome	Rose or red macules, urticarial plaques	IgM (few have IgG component) [35]
Necrobiotic xanthogranuloma	Waxy, yellow, plaques, nodules	IgG (kappa) [36]
POEMS syndrome	Hyperpigmentation, glomeruloid hemangioma	IgA or IgG (lambda) [37]
Pyoderma gangrenosum	Pustules, ulcerated plaques	IgA [38]
Cold agglutinin disease	Livido reticularis, raynaud phenomenon, acrocyanosis, ulceration	IgM (kappa) (few have IgA, few polyclonal) [39]
Papular mucinosis	Small, generally localized, lichenoid papular lesions	IgG (lambda) [30]
Subcorneal pustular dermatosis	Vesiculopustular eruptions	IgA [40]
Erythema elevatum diutinum	Plaques, nodules often localized to extensor surfaces	IgA [41]
Scleredema	Thickened, indurated plaques, most often affecting trunk	IgG and IgA [42]

## Abbreviations

ACL: Acquired cutis laxa
CL: Cutis laxa
CRP: C-reactive protein
CyBorD: Cyclophosphamide, bortezomib, and dexamethasone
ESR: Erythrocyte sedimentation rate
LHCDD: Light and heavy chain deposition disease
MGODS: Monoclonal gammopathy of dermatological significance
MGUS: Monoclonal gammopathy of undetermined significance
POEMS: Polyneuropathy, organomegaly, endocrinopathy, monoclonal protein, and skin changes
PET-CT: Positron emission tomography-computed tomography.
